# Guard cell SLAC1‐type anion channels mediate flagellin‐induced stomatal closure

**DOI:** 10.1111/nph.13435

**Published:** 2015-04-30

**Authors:** Aysin Guzel Deger, Sönke Scherzer, Maris Nuhkat, Justyna Kedzierska, Hannes Kollist, Mikael Brosché, Serpil Unyayar, Marie Boudsocq, Rainer Hedrich, M. Rob G. Roelfsema

**Affiliations:** ^1^ Molecular Plant Physiology and BiophysicsJulius‐von‐Sachs Institute for BiosciencesBiocenterUniversity of WürzburgJulius‐von‐Sachs‐Platz 2D‐97082WürzburgGermany; ^2^ Faculty of Science and LettersDepartment of BiologyUniversity of Mersin33343MersinTurkey; ^3^ Institute of TechnologyUniversity of TartuNooruse 1Tartu50411Estonia; ^4^ Division of Plant BiologyDepartment of BiosciencesUniversity of HelsinkiPO box 65FI‐00014HelsinkiFinland; ^5^ Institute of Plant Sciences Paris‐SaclayUMR9213/UMR1403 CNRS‐INRA‐Université Paris Sud‐Université Evry Val d'Essonne‐Université Paris DiderotSaclay Plant SciencesBat 630, rue Noetzlin91405OrsayFrance

**Keywords:** ABA, *Arabidopsis thaliana*, flg22, guard cells, innate immunity, microbe‐associated molecular pattern (MAMP), S‐type anion channel, stomata

## Abstract

- During infection plants recognize microbe‐associated molecular patterns (MAMPs), and this leads to stomatal closure. This study analyzes the molecular mechanisms underlying this MAMP response and its interrelation with ABA signaling.
- Stomata in intact *Arabidopsis thaliana* plants were stimulated with the bacterial MAMP flg22, or the stress hormone ABA, by using the noninvasive nanoinfusion technique. Intracellular double‐barreled microelectrodes were applied to measure the activity of plasma membrane ion channels.
- Flg22 induced rapid stomatal closure and stimulated the SLAC1 and SLAH3 anion channels in guard cells. Loss of both channels resulted in cells that lacked flg22‐induced anion channel activity and stomata that did not close in response to flg22 or ABA. Rapid flg22‐dependent stomatal closure was impaired in plants that were flagellin receptor (FLS2)‐deficient, as well as in the *ost1‐2* (*Open Stomata 1*) mutant, which lacks a key ABA‐signaling protein kinase. By contrast, stomata of the ABA protein phosphatase mutant *abi1‐1* (*ABscisic acid Insensitive 1*) remained flg22‐responsive.
- These data suggest that the initial steps in flg22 and ABA signaling are different, but that the pathways merge at the level of OST1 and lead to activation of SLAC1 and SLAH3 anion channels.

## Introduction

Pathogenic bacteria take advantage of stomatal pores in the leaf surface to enter their host plants. During infection, plant cells recognize microbe‐associated molecular patterns (MAMPs), such as flagellin or chitosan, and counteract the microbial invasion by stomatal closure (Melotto *et al*., 2006, 2008; Schulze‐Lefert & Robatzek, 2006; Boller & Felix, 2009). In guard cells of barley, chitosan triggers the activation of S‐type anion channels and induces stomatal closure (Koers *et al*., 2011). Likewise, the drought hormone ABA causes stomatal closure, by activating the S‐type anion channels, slow anion channel 1 (SLAC1) and SLAC1 homolog 3 (SLAH3) (Roelfsema *et al*., 2012). Because of these similarities in responses to ABA and MAMPs, we compared stomatal responses induced by both stimuli and studied the signaling pathways that target the SLAC1 and SLAH3 anion channels.

Nonhost defense responses occur early after infection and are triggered by binding of MAMPs to plasma membrane receptor‐like kinases (RLKs) or receptor‐like proteins (RLPs) (Boller & Felix, 2009). One of the best studied RLKs is the FLagellin Sensing 2 (FLS2) receptor of *Arabidopsis thaliana*, which can recognize a 22‐amino‐acid residue stretch of the flagellin protein from *Pseudomonas syringae* (flg22) (Felix *et al*., 1999; Gomez‐Gomez & Boller, 2000; Sun *et al*., 2013). Upon binding of flg22, FLS2 forms a complex with a second RLK, BRI1‐associated kinase 1 (BAK1), or one of its somatic embryogenesis‐related kinase (SERK) homologs (Chinchilla *et al*., 2007, 2009; Ranf *et al*., 2011). The early signaling phase of nonhost resistance is characterized by a number of responses, occurring within 5 min after stimulation with MAMPs (Boller & Felix, 2009). In several cell types, this phase has been associated with a transient depolarization of the plasma membrane (Pelissier *et al*., 1986; Kuchitsu *et al*., 1997; Jeworutzki *et al*., 2010). Membrane depolarization in mesophyll cells is associated with a rise in the cytosolic free Ca^2+^ concentration (Blume *et al*., 2000; Lecourieux *et al*., 2005; Hedrich, 2012) and activation of NADPH oxidases that produce reactive oxygen species (ROS) (Nurnberger *et al*., 1994; Torres *et al*., 2002; Hedrich, 2012).

Guard cells respond to various abiotic (Hetherington & Woodward, 2003) and biotic stress conditions, but the extent to which these responses are based on the same molecular machinery is unknown. Microbial invasions are recognized via MAMPs such as flg22, whereas drought evokes changes in the cytosolic concentration of the stress hormone ABA. An overlap of both signaling pathways can be expected, as the ABA‐insensitive *ost1* and *ost2* (*Open Stomata 1* and *2*) mutants were reported to lack responses to flg22 (Melotto *et al*., 2006; Liu *et al*., 2009). However, the direct role of OST1 in the guard cell response to flg22 was questioned by a later study (Montillet *et al*., 2013). With respect to ABA responses, the function of the OST1 protein kinase in guard cells is firmly established. OST1 phosphorylates and activates the anion channel SLAC1 (Geiger *et al*., 2009; Lee *et al*., 2009; Vahisalu *et al*., 2010), whereas a second S‐type anion channel, SLAH3, is activated by calcium‐dependent protein kinases (CPKs) in an OST1‐dependent manner (Geiger *et al*., 2011; Demir *et al*., 2013). In contrast to the ABA responses, little is known about targeting of guard cell anion channels by flg22.

Molecular mechanisms that mediate flg22‐induced stomatal closure and their overlap with the ABA‐signaling chain were studied with a combination of two microcapillary‐based techniques. First, the noninvasive nanoinfusion technique was applied (Hanstein & Felle, 2004; Koers *et al*., 2011), in which microcapillaries are guided through open stomata to stimulate stomata in intact plants with flg22 and ABA. Second, intracellular double‐barreled microelectrodes were used to monitor the ion channel activity of single guard cells. These studies showed that flg22 activates the anion channels SLAC1 and SLAH3 and that these channels are required for rapid stomatal closure. Based on the results with several signaling mutants, we found that MAMP‐ and ABA‐signaling pathways meet at the level of the protein kinase OST1.

## Materials and Methods

### Plant growth and material

Seeds of *Arabidopsis thaliana* (L.) Heynh. were sown on sterilized soil and grown in a climate cabinet with a 12 : 12 h, 22 : 16°C, day : night cycle and a photon flux density of 150 μmol m^−2^ s^−1^. After 14 d, the seedlings were transferred to cut‐off centrifuge tubes (diameter 27 mm), filled with sterilized soil and grown under the same conditions as before. The mutants *ost1‐2* and *abi1‐1* (*ABA Insensitive 1‐1*) were in the Landsberg *erecta* (L*er*) background, whereas *cpk3/5/6/11*,* rbohD/F* (*Respiratory Burst Oxidase Homologs‐D/F*), *slac1‐3* and *slah3‐1* were in *Columbia 0* (Col‐0) and FLS2 was expressed in *Wassilewskija* (Ws‐0) (Zipfel *et al*., 2004). The *slac1‐3* (SALK_099139) and *slah3‐1* (GK‐371G03) seeds were obtained from the Nottingham Arabidopsis Stock Centre (http://arabidopsis.info/). The *slac1‐3/slah3‐1* double mutant was obtained through standard crossing and genotyping in the F2 and F3 generation using slac1–3 LP AACTTCTTCTTCGCTCCTTGG, slac1‐3 RP GACCATTTCTTTGCCTGTTTG, Salk Lba TGGTTCACGTAGTGGGCCATCG, LB‐GK2 CCCATTTGGACGTGAATGTAGACAC, SLAH3 C‐terminus‐rev GGATAATGGTGGTCACGAGCAG, and SLAH3 N‐terminus‐for ACCCCATTTCCACCTTCGGTATG. The *cpk3/5/6/11* quadruple mutant was obtained by crossing *cpk3* (SALK_022862; Mori *et al*., 2006) and *cpk5/6/11* (Boudsocq *et al*., 2010). The quadruple mutant was genotyped with the following primers: for the Salk lines, LBb salk GCGTGGACCGCTTGCTGCAACT with CPK3 LP TCACCTGCATTAATGGATCAAC and CPK3 RP GTCCACCATGTTAAACCTGCC, CPK6 LP CTCGCAACTAACGCTTACCTG and CPK6 RP TTTTGGGATCTATAATGATCGATG, CPK11 LP AAATGATGGTGTTTTTATTTATGTAAAG and CPK11 RP AAACCAATTAGGCGATGAACC; for the sail line, LBa sail TTCATAACCAATCTCGATACAC and CPK5 LP TCGTTCCAAATTGACCTTGAC and CPK5 RP GAGGAAACAGCGGAGAGAGAC.

### Reverse transcription polymerase chain reaction (RT‐PCR) analysis

Total RNA was isolated from 10‐d‐old seedlings of Col‐0 and the *cpk3/5/6/11* mutant using the NucleoSpin® RNA II kit (Macherey‐Nagel, Düren, Germany). cDNA was synthesized from 1 μg of total RNA using 0.5 μg of oligo(dT) primer and SSII reverse transcriptase (Life Technologies, Carlsbad, CA, USA). RT‐PCR analysis was carried out with the following primers: CPK3‐for GACACAGCAAGTCCAAATC and CPK3‐rev AACTGGAATGCGGTGTAC; CPK5‐for GACGAAGGCGATAACAATAA and CPK5‐rev CCGCTCTAGTTTGTTGAGAT; CPK6‐for AAATCCACCACCACTACTGT and CPK6‐rev ACTGAAATGCAGAGACAAGAT; and CPK11‐for GAGACGAAGCCAAACCCTA and CPK11‐rev GCTGTAAACTCCGAGAAATC. *CPK4* was used as a control and amplified with primers CPK4‐for TCCATACGAAACACCAAGA and CPK4‐rev GTTCCTCATAGTTCTGCTCC.

### Nanoinfusion experiments

Plants aged 5–6 wk old were mounted into a holder on the microscope table of an upright microscope (Axioskop 2FS, Zeiss, Jena, Germany). The plants were tilted and a fully grown leaf was attached with its adaxial side to an acrylic glass block, using double‐sided adhesive tape. Stomata on the abaxial side of the epidermis were visualized with a water immersion objective (Achroplan ×40/0.8 W, Zeiss) and a drop of bath solution A (5 mM KCl, 0.1 mM CaCl_2_ and 5 mM K/citrate pH 5) was placed between the objective and the leaf surface. Images were captured with a camera (IMAG‐K4, Walz, Effeltrich, Germany) at an interval of 0.5 s using Kappa Cameras software (Kappa Camera Control, Kappa, Gleichen, Germany).

Nanoinfusion (Hanstein & Felle, 2004; Koers *et al*., 2011) was carried out with borosilicate glass capillaries (inner/outer diameter = 0.56/1.0 mm; Hilgenberg, Malsfeld, Germany), pulled on a horizontal laser puller (P2000, Sutter Instrument, Novato, CA, USA). The tip was broken to obtain an opening of *c*. 1 μm. The microcapillaries were backfilled, either with control solution containing 10 mM K/Mes (2‐(N‐morpholino) ethanesulfonic acid), pH 6, 1 mg ml^−1^ BSA and 2 × 10^−3^% v/v dimethyl sulfoxide, or with the same solution including 10 μM ABA or 20 nM flg22. The capillaries were manipulated into an open stoma with a micromanipulator (MHW‐3, Narishige, Tokyo, Japan). Before nanoinfusion, stomata were monitored for at least 10 min, to exclude stomatal closure induced by mechanical stimulation. Solutions were injected into the apoplast by applying a back‐pressure of 140 kPa for 0.1–2.5 s, using a pneumatic drug injector (PEDS‐02; NPI Electronic, Tamm, Germany). Successful nanoinfusion was evident from changes in the transparency of cells in the epidermis.

### Microelectrode measurements

Guard cells were impaled with double‐barreled microelectrodes in intact plants as well as epidermal peels. Leaves of intact plants were fixed on the microscope table as described earlier for the nanoinfusion procedure. Epidermal peels were gently peeled from the abaxial side of *Arabidopsis* leaves with tweezers and attached to microscope slides with medical adhesive (Medical Adhesive B, Aromando, Düsseldorf, Germany). Directly after isolation of the epidermis, the microscope slides were transferred to Petri dishes with bath solution B (50 mM KCl, 0.05 mM CaCl_2_, and 1 mM K/bis‐TRIS propane (BTP), pH 6.0). Stomatal opening was induced with white light at a photon flux density of 150 μmol m^−2^ s^−1^. Before measurement, the microscope slides were mounted into measuring chambers that were filled with bath solution C (50 mM KCl, 1 mM CaCl_2_ and 1 mM BTP, pH 6.0). Mesophyll tissue was obtained by cutting a rosette leaf along its central vein. After attaching the adaxial side of the leaf tissue to double‐sided adhesive tape, the abaxial epidermis was gently removed with tweezers, thereby gaining access to the spongy mesophyll. The tissue was rapidly transferred to bath solution D (5 mM KCl, 1 mM CaCl_2_, and 10 mM Mes/BTP, pH 6.0) and allowed to recover from the isolation procedure for at least 12 h.

Mesophyll cells and guard cells were impaled with single‐ and double‐barreled microelectrodes, respectively. All electrodes were pulled from the same glass capillaries as used for nanoinfusion. For producing double‐barreled electrodes, two capillaries were aligned, heated, and twisted by 360° and pre‐pulled on a customized vertical electrode puller (L/M‐3PA, HEKA, Lambrecht, Germany). Sharp tips of single‐ and double‐barreled electrodes were made on a horizontal laser puller (P2000, Sutter), the electrodes were backfilled with 300 mM KCl or 300 mM CsCl and had resistances ranging from 60 to 100 MΩ for single barreled electrodes and 160 to 240 MΩ for double barreled electrodes. The electrodes were connected with Ag/AgCl half‐cells to head stages (HS‐2A × 0.01; Axon Instruments, Molecular Devices, Sunnyvale, CA, USA) of two‐electrode voltage clamp amplifiers (Axoclamp 2B or Geneclamp 500; Axon Instruments). The reference electrode consisted of a glass capillary filled with 300 mM KCl and plugged with 2% w/v agarose in 300 mM KCl, connected to ground by an Ag/AgCl half‐cell. Pulse protocols were applied with patch clamp software (Pulse, HEKA) and data were sampled at 1 or 0.1 kHz and filtered with a low‐pass Bessel filter at 200 or 20 Hz. Microelectrodes were impaled into the cells of interest, with a lightweight piezo‐driven micromanipulator (MM3A; Kleindiek, Reutlingen, Germany).

### Amperometric hydrogen peroxide (H_2_O_2_) detection

The production of H_2_O_2_ by mesophyll cells was monitored based on the method described previously by Amatore *et al*. (2006). Measurements were carried out with a platinum‐iridium electrode (MicroProbes, Gaithersburg, MD, USA), which was cut back to a disc of *c*. 100 μm in diameter. The platinum‐iridium disc was gently placed in close proximity to mesophyll cells (MHW‐3, Narishige) and held to a constant voltage of 600 mV with an amperometry amplifier (VA 10X, NPI Electronic). Oxidation of H_2_O_2_ at the tip of a platinum‐iridium microelectrode resulted in a current signal, which was low‐pass‐filtered at 20 Hz and recorded with patch master software (Patch Master, HEKA). The electrode was calibrated in freshly prepared bath solutions with defined H_2_O_2_ concentrations (Supporting Information Fig. S1a,b). Flg22‐induced currents at the electrode were interpreted as H_2_O_2_ production, based on the observation that the addition of 6050 U ml^−1^ catalase prevented the stimulus‐induced current signal and no flg22‐induced signals were observed with the *Atrboh*D mutant (Fig. S1c).

## Results

### Pathogen‐derived flg22 induces rapid stomatal closure

**Figure 1:**
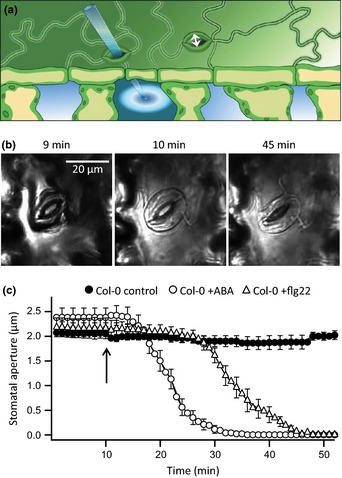
Nanoinfusion of flg22 and ABA triggers rapid stomatal closure in intact *Arabidopsis* leaves. (a) Illustration of the nanoinfusion technique used to induce flg22‐ and ABA‐dependent stomatal closure. A microcapillary was moved into the substomatal cavity of an open stoma and used to infuse solutions into the intercellular space. Movement of neighboring stomata was monitored on an upright microscope. (b) Images of a stoma in the abaxial epidermis of an *Arabidopsis* leaf stimulated by nanoinfusion of 20 nM flg22. Images were obtained just before (left panel), directly after (middle panel), and 35 min after stimulation with flg22 (right panel). Note that the leaf becomes transparent because of solution infused into the intercellular space. (c) Time‐dependent changes in average stomatal aperture before and after stimulation with control solution (closed circles, *n* = 8), 10 μM ABA (open circles, *n* = 13), or 20 nM flg22 (open triangles, *n* = 13); the arrow indicates the time point of nanoinfusion. Data are presented as average values of 8 to 13 stomata of at least three independent experiments, and error bars represent ± SE.

Stomatal responses to flg22 were studied with the nanoinfusion technique (Hanstein & Felle, 2004; Koers *et al*., 2011), in which a microcapillary (opening *c*. 1 μm) is positioned through an open stoma in its substomatal cavity (Fig. 1a). A solution can be infused into the intracellular spaces of the leaf by pressure injection. The onset of nanoinfusion was evident by a change in optical properties of the leaf surface, causing epidermal cells and guard cells to appear more transparent with better visibility (Fig. 1b). Stomata within these leaf patches were monitored for agonist‐induced, time‐dependent changes in aperture.

The FLS2 receptor has a high affinity for flg22 and saturates at concentrations as low as 10 nM (Felix *et al*., 1999; Jeworutzki *et al*., 2010). We therefore used a concentration of 20 nM flg22, to fully stimulate FLS2 receptors, while preventing unspecific peptide responses. In wild‐type leaves, nanoinfusion of flg22 triggered fast stomatal closure after a lag time of 17 min (SE = 1, *n* = 13) (Fig. 1b,c; Movie S1). As a reference, stomata were also stimulated with the stress hormone ABA, which is fully active at a concentration of 10 μM (Roelfsema & Prins, 1995; Roelfsema *et al*., 2006). Nanoinfusion of ABA triggered a very similar response to that of flg22, but the lag time was much shorter (7 min, SE = 1, *n* = 13) (Fig. 1b,c). Even though the flg22‐ and ABA‐induced responses differed in lag time, stomatal closure occurred with the same maximal velocity: 0.24 μm min^−1^ (SE = 0.04, *n* = 13) and 0.26 μm min^−1^ (SE = 0.02, *n* = 13) for flg22 and ABA, respectively.

### Flg22 inhibits K^+^ uptake channels and activates S‐type anion channels

**Figure 2:**
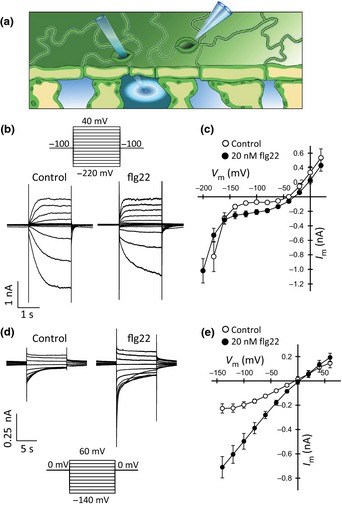
Nanoinfusion of flg22 stimulates S‐type anion channels in guard cells. (a) Illustration of the nanoinfusion technique, combined with the voltage‐clamp technique, using intracellular double‐barreled microelectrodes in a guard cell of a neighboring stoma. (b) Guard cells of *Arabidopsis thaliana* acc. Landsberg *erecta* in intact plants were impaled with double‐barreled microelectrodes, filled with 300 mM KCl, and clamped at a holding potential of −100 mV. A bipolar step protocol (upper panel) was used to obtain current traces within 30 min after nanoinfusion of control solution (left lower panel) or 20 nM flg22 (right lower panel). (c) Current–voltage plots of guard cells clamped from a holding potential of −100 mV to test pulses ranging from −200 to 20 mV, as shown in (b). Data are presented as average values of eight guard cells, and error bars represent ± SE. (d) K^+^ efflux channels were blocked by filling electrodes with 300 mM CsCl and clamping guard cells to a holding potential of 0 mV. A bipolar step protocol (lower panel) was used to obtain current traces within 30 min after nanoinfusion of control solution (left upper panel) or 20 nM flg22 (right upper panel). (e) Current–voltage relationships for guard cells clamped from a holding potential of 0 mV to test pulses ranging from 60 to −140 mV, as shown in (d). Data are presented as average values of 10 guard cells, and error bars represent ± SE.

Stomatal closure in general, and ABA‐induced closure in particular, is provoked by the extrusion of K^+^ salts from guard cells (Roelfsema & Hedrich, 2005). The impact of flg22 on ion transport was therefore tested by impaling guard cells in intact leaves with double‐barreled microelectrodes. Guard cells were first stimulated by nanoinfusion of 20 nM flg22 through an open stoma, followed by impalement of a guard cell in a neighboring stoma (Fig. 2a). In experiments with KCl‐filled electrodes, plasma membrane K^+^ efflux channels gave rise to slowly emerging outward currents at positive voltages, whereas the activity of K^+^ uptake channels facilitates slowly activating inward currents at most negative voltages (Fig. 2b,c) (Roelfsema & Hedrich, 2005; Kollist *et al*., 2014). Stimulation with flg22 weakly inhibited these K^+^ uptake channels, whereas the activity of K^+^ efflux channels was unaffected. These results are in line with a previous study carried out with the patch clamp technique (Zhang *et al*., 2008).

Nanoinfusion of flg22 also caused an increase of the inward current at potentials ranging from −140 to −60 mV (Fig. 2c). In this voltage range, K^+^‐selective uptake and efflux channels are not active (Roelfsema & Prins, 1997) and the inward currents are therefore most likely to be carried by plasma membrane anion channels. To test this hypothesis, the activity of anion channels was measured with microelectrodes filled with Cs^+^, thereby inhibiting K^+^ efflux channels. The guard cell plasma membrane was clamped from a holding potential of 0 mV, with block pulses ranging from 60 to −140 (Fig. 2d). Under these conditions, the plasma membrane conductance was dominated by S‐type anion channels, which slowly deactivate and conduct inward currents at the most negative test potentials (Linder & Raschke, 1992; Vahisalu *et al*., 2008; Geiger *et al*., 2009). Nanoinfusion of 20 nM flg22 stimulated the activity of these S‐type anion channels in guard cells (Fig. 2d,e).

### Flg22 stimulates the S‐type anion channels SLAC1 and SLAH3

**Figure 3:**
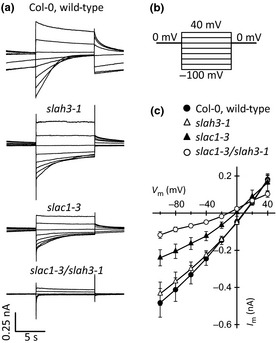
Both SLAC1 and SLAH3 contribute to the S‐type anion conductance of guard cells. (a) Guard cells of *Arabidopsis thaliana* acc. Columbia 0 (Col‐0) were impaled with double‐barreled electrodes filled with 300 mM CsCl and clamped to a holding potential of 0 mV. Experiments were carried out with epidermal strips to ensure identical extracellular ion concentrations for all guard cells. (b) Bipolar step protocol used to obtain current traces of the wild‐type, and the *slac1‐3*,* slah3‐1*, and *slac1‐3*/*slah3‐1* loss‐of‐function mutants as shown in (a). (c) Current–voltage relationship for guard cells of Col‐0 (closed circles), *slah3‐1* (open triangles), *slac1‐3* (closed triangles), and *slac1‐3/slah3‐1* (open circles). Data are presented as average values of eight to 11 cells, and error bars represent ± SE.

Guard cell S‐type anion channels are encoded by *SLAC1* and *SLAH3* (Negi *et al*., 2008; Vahisalu *et al*., 2008; Geiger *et al*., 2011) and represent key targets for ABA‐induced stomatal closure (Geiger *et al*., 2009, 2011; Lee *et al*., 2009; Roelfsema *et al*., 2012). However, the relative contribution of both genes to the S‐type anion channel conductance of guard cells has not yet been resolved. To evaluate the impact of flg22 signaling on S‐type anion channels in guard cells, we isolated single loss‐of‐function mutants for *SLAC1* and *SLAH3* and generated the double mutant (Fig. 3). Guard cells in epidermal strips were impaled with electrodes filled with CsCl to block currents carried by K^+^ efflux channels. Under these conditions, a high activity of S‐type anion channels was measured when stimulating guard cells with voltage pulses from a holding potential of 0 mV to test voltages ranging from 40 to −100 mV (Fig. 3a,b).

Loss of SLAH3 function did not significantly alter the conductance of the S‐type anion channels, but S‐type anion currents were clearly reduced in *slac1‐3* (Fig. 3a,c). This suggests a major role for SLAC1, which is in line with its important function in stomatal closure (Negi *et al*., 2008; Vahisalu *et al*., 2008; Roelfsema *et al*., 2012). However, S‐type anion currents were only abolished in the *slac1‐3/slah3‐1* double mutant (Fig. 3a,c), showing that both the SLAC1 and SLAH3 channels contribute to the anion conductance of *Arabidopsis* guard cells. The contribution of both channels is not simply additive, which may be a result of post‐translational regulation of SLAC1 and SLAH3 by protein kinases (Geiger *et al*., 2009, 2011; Lee *et al*., 2009).

**Figure 4:**
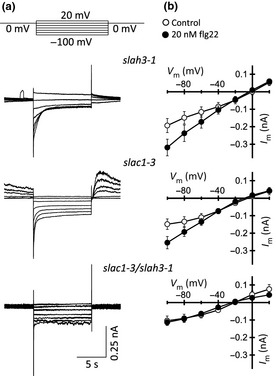
Flg22 stimulates the activity of SLAC1, as well as SLAH3. (a) Guard cells of intact *Arabidopsis thaliana* acc. Col‐0 plants were impaled with double‐barreled electrodes, filled with 300 mM CsCl, and clamped from a holding potential of 0 mV. Bipolar step protocols (upper panel) were applied to obtain current traces for the single *slah3‐1* and *slac1‐3* loss‐of‐function mutants, as well as the *slac1–3*/*slah3–1* double mutant. Data were obtained within 30 min after stimulation by nanoinfusion with flg22. (b) Current–voltage relationship for guard cells stimulated by nanoinfusion with control solution (open circles) or 20 nM flg22 (closed circles), obtained with pulse protocols as shown in (a). Data are presented as average values of eight cells, and error bars represent ± SE.

**Figure 5:**
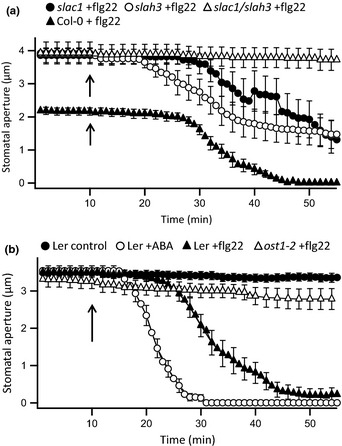
The S‐type anion channels SLAC1 and SLAH3, and the protein kinase OST1 are essential for rapid flg22‐induced stomatal closure. (a) Time‐dependent stomatal movement induced by nanoinfusion of 20 nM flg22 in *slac1‐3* (closed circles, *n* = 9), *slah3‐1* (open circles, *n* = 16), *slac1‐3*/*slah3‐1* (open triangles, *n* = 11), and wild‐type Col‐0 (closed triangles, data from Fig. 1c). Data are given as average values of nine to 16 stomata from at least four independent experiments, and error bars represent ± SE; arrows indicate the time point of nanoinfusion. (b) Time‐dependent stomatal movement of *Arabidopsis thaliana* acc. Landsberg *erecta* (L*er*) stimulated by nanoinfusion of control solution (closed circles, *n* = 20), 10 μM ABA (open circles, *n* = 20), and 20 nM flg22 (closed triangles, *n* = 21), as well as the response of the *ost1‐2* mutant to flg22 (open triangles, *n* = 17). Data are given as average values of 17–21 stomata from at least four independent experiments, and error bars represent ± SE.

Because of the presence of two S‐type anion channels in guard cells, we tested the relative contribution of SLAC1 and SLAH3 to the flg22‐induced increase in anion channel conductance. Guard cells in intact plants of the single *slac1‐3* and *slah3‐1* mutants, as well as the double mutant, were impaled after nanoinfusion with 20 nM flg22 or a control solution (Fig. 4a). As in epidermal strips, slowly deactivating S‐type channels were detected in the *slah3‐1* and *slac1‐3* single mutants, but not in the *slac1–3/slah3‐1* double mutant (Fig. 4a). Flg22 was able to activate S‐type anion channels in both single mutants, but the *slac1‐3/slah3‐1* double mutant was insensitive to MAMP stimulation (Fig. 4b). The stomata of all three S‐type channel loss‐of‐function mutants were more open in comparison to the Col‐0 wild‐type (Fig. 5a). Nanoinfusion of 20 nM flg22 into leaves of *slac1‐3* and *slah3‐1* resulted in stomatal closure, but the stomata did not close completely (Fig 5a). Loss of both the SLAC1 and SLAH3 channels resulted in stomata that were completely insensitive to flg22 stimulation (Fig 5a).

### The flg22‐ and ABA‐signaling pathways merge at OST1

Flg22 targets the same anion channels in guard cells as previously shown for ABA (Geiger *et al*., 2009, 2011; Lee *et al*., 2009; Brandt *et al*., 2012; Scherzer *et al*., 2012), suggesting that both stimuli evoke very similar responses. The protein kinase OST1 plays a central role in guard cell ABA signaling (Joshi‐Saha *et al*., 2011), but its role in flg22 responses is controversially discussed (Melotto *et al*., 2006; Montillet *et al*., 2013). We therefore compared the responses of *ost1‐2* stomata to ABA and flg22 with the L*er* wild‐type in which this mutant was isolated, using the nanoinfusion technique (Mustilli *et al*., 2002). In intact leaves, *ost1‐2* stomata did not close in response to ABA (data not shown) or to flg22 (Fig. 5b). It appears that OST1 is essential for rapid stomatal closure triggered by 10 μM ABA, as well as 20 nM flg22.

OST1 can activate SLAC1 by phosphorylation of its N‐terminal domain (Geiger *et al*., 2009; Vahisalu *et al*., 2010), but it is unable to stimulate SLAH3 (Geiger *et al*., 2011). By contrast, both channels can be activated by several CPKs (CPK3, 6, 21 and 23), as well as by CIPK23 (Geiger *et al*., 2010, 2011; Brandt *et al*., 2012; Scherzer *et al*., 2012; Demir *et al*., 2013; Maierhofer *et al*., 2014). CPKs (CPK4, 5, 6 and 11) are also involved in the flg22‐induced production of ROS by NADPH oxidases (Boudsocq *et al*., 2010; Dubiella *et al*., 2013). Based on current knowledge, a link between CPKs, ROS production, and the flg22‐induced activation of anion channels is to be expected.

**Figure 6:**
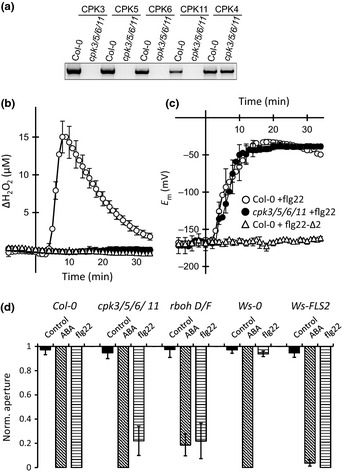
Calcium‐dependent protein kinases 3 (CPK3), 5, 6, and 11 are essential for the flg22‐induced reactive oxygen species (ROS) production of mesophyll cells, but not for their depolarization or for stomatal closure. (a) Expression level of *CPKs* in wild‐type *Arabidopsis thaliana* acc. Col‐0 and the *cpk3/5/6/11* mutant, analyzed by reverse transcription polymerase chain reaction (RT‐PCR). The expression of *CPK3*,*CPK5*,*CPK6*, and *CPK11* is abolished in the quadruple mutant, while that of the control *CPK4* is not affected. (b) Hydrogen peroxide (H_2_O_2_) production of mesophyll tissue measured with a platinum microdisc electrode in Col‐0 (open circles) and the *cpk3/5/6/11* mutant (closed circles) stimulated with flg22, as well as Col‐0 exposed to flg22‐Δ2 (open triangles). Error bars represent ± SE of six experiments. (c) Membrane potential recordings of mesophyll cells of Col‐0 (open circles) and *cpk3/5/6/11* (closed circles) stimulated with flg22, as well as Col‐0 exposed to flg22‐Δ2 (open triangles). Error bars represent ± SE of six experiments. (d) Normalized data of stomatal closure, induced by nanoinfusion of control solution, 10 μM ABA, or 20 nM flg22. The stomatal apertures at the start of the experiments were set to 1 and final apertures, measured after 40 min for ABA responses or 50 min for flg22 and control experiments, are shown relative to the starting values. Data are shown for the *cpk3/5/6/11* quadruple mutant, the *rboh*‐D/F double loss‐of‐function mutant, Wassilewskija (Ws‐0), and Ws‐0 transformed with a functional FLS2 receptor. In *cpk3/5/6/11*, three out of 16 stomata did not respond to flg22, whereas in *rbohD/F*, two out of 11 and four out of 12 stomata did not close in response to flg22 and ABA, respectively. Data are given as normalized values (aperture before nanoinfusion = 1) of at least eight experiments. Error bars represent ± SE.

Because of the functional redundancy among CPKs, we generated a quadruple loss‐of‐function mutant of CPK3, 5, 6 and 11 (Fig. 6a). The ability of this mutant to produce ROS in response to flg22 was tested with a platinum‐iridium disc electrode, which had a linear dependency on the H_2_O_2_ concentration (Fig. S1a,b). In wild‐type mesophyll tissue, the electrode detected an increase in ROS concentration, equal to 15 μM H_2_O_2_, *c*. 10 min after flg22 application (Fig. 6b). The ROS signal was likely to be produced by plasma membrane‐bound RBOH*D* proteins, as no flg22‐dependent ROS production was recorded for the *rboh*D mutant (Fig. S1c). Furthermore, this ROS production and the associated depolarization are specific for flg22, as these responses are not triggered by the inactive flg22‐Δ2 peptide, which lacks two amino acids at the C‐terminus (Fig. 6c) (Bauer *et al*., 2001). In contrast to the Col‐0 wild‐type, mesophyll cells of the *cpk3/5/6/11* quadruple mutant did not produce ROS in response to flg22 (Fig. 6b). Nonetheless, the MAMP was capable of inducing a depolarization of *cpk3/5/6/11* mesophyll cells (Fig. 6c), just as in the wild‐type (Jeworutzki *et al*., 2010). This suggests that CPK3, 5, 6 and 11 are required for flg22‐induced ROS production, but not for the flg22‐induced depolarization of mesophyll cells.

The role of the four CPKs in guard cell signaling was tested by nanoinfusion of flg22. In the *cpk3/5/6/11* mutant, 13 out of 16 stomata closed in response to flg22 application (Figs 6d, S2a). We also tested the double loss‐of‐function mutant of the NADPH oxidases *AtRBOH*D and F, which have been implicated in ABA and flg22 responses of guard cells (Kwak *et al*., 2003; Macho *et al*., 2012). Nanoinfusion of flg22 caused closure of nine out of 11 stomata of *rbohD/F*, while eight out of 12 stomata were ABA‐responsive (Figs 6d, S2b). This shows that neither the loss of CPK3/5/6/11 nor that of RBOH*D/F* causes complete loss of sensitivity to flg22 and ABA.

The guard cell signaling chains, activated by flg22 and ABA, share OST1 and seem not to differ with respect to downstream components. However, flg22 and ABA‐dependent signaling are likely to diverge upstream of OST1, suggested by the variation in response time (Figs 1c, 5b) and the nature of their receptors. Whereas the ABA receptor complex resides within the cytosol (Ma *et al*., 2009; Park *et al*., 2009), flg22 is recognized in the extracellular space by the FLS2 receptor (Gomez‐Gomez & Boller, 2000). The FLS2 receptor is not functional in the naturally occurring Ws‐0 accession (Gomez‐Gomez *et al*., 1999) and we tested the stomata of this accession by nanoinfusion. Stomata of Ws‐0 did not close after nanoinfusion of flg22, but did display ABA sensitivity (Figs 6d, S2c). Complementation of Ws‐0 with a functional FLS2 receptor (Zipfel *et al*., 2004) renders stomata sensitive to flg22 (Figs 6d, S2c). This shows that the FLS2 receptor is essential for the rapid flg22‐induced stomatal closure, but not for ABA responses.

**Figure 7:**
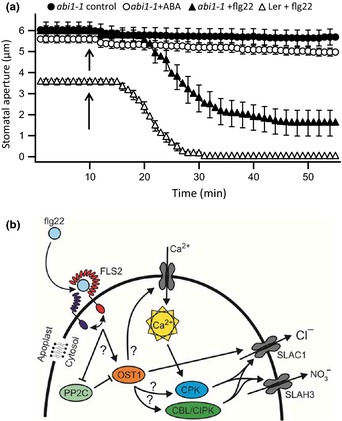
Abscisic acid and flg22 signaling pathways merge at OST1. (a) Time‐dependent stomatal movement of the *Arabidopsis thaliana* acc. Landsberg *erecta* (L*er*) *abi1‐1* mutant, stimulated by nanoinfusion of control solution (closed circles, *n* = 11), 10 μM ABA (open circles, *n* = 14), and 20 nM flg22 (closed triangles, *n* = 13); for comparison, data of the L*er* wild‐type stimulated with flg22 (open triangles, *n* = 21) from Fig. 5(b) are shown. Data are given as average values of 11–14 stomata from at least four independent experiments, and error bars represent ± SE; arrows indicate the time point of nanoinfusion. (b) Schematic representation of the signaling pathway for flg22‐induced membrane responses in guard cells. Flg22 binds to receptor‐like kinase FLS2 in the plasma membrane, which interacts with the BRI1‐associated kinase 1 (BAK1) and somatic embryogenesis‐related kinase (SERK) coreceptors. The interaction between both receptors leads to OST1 activation, either through the inhibition of PP2Cs or by an alternative mechanism. OST1 can directly phosphorylate and activate SLAC1, which releases anions into the guard cell wall. OST1 may activate Ca^2+^‐permeable channels, causing a cytosolic Ca^2+^ signal. The cytosolic Ca^2+^ signal will activate calcium‐dependent protein kinases (CPKs), which can activate SLAC1, as well as SLAH3. Likewise, Ca^2+^ can bind to calcineurin B‐like (CBL) proteins that interact with CBL‐interacting protein kinases (CIPKs), which in turn can activate SLAC1 and SLAH3. Alternatively, OST1 could be capable of activating CPKs or CIPKs through a Ca^2+^‐independent mechanism.

In the ABA signaling pathway, OST1 is inhibited by a class of type 2C protein phosphatases (Cutler *et al*., 2010; Raghavendra *et al*., 2010), including ABI1. In the *abi1‐1* mutant (Koornneef *et al*., 1984), ABI1 constitutively inhibits OST1 (Gosti *et al*., 1999; Yoshida *et al*., 2006; Vlad *et al*., 2009) and prevents the ABA‐induced activation of SLAC1 (Geiger *et al*., 2009; Lee *et al*., 2009) and SLAH3 (Geiger *et al*., 2011). Consequently, the stomatal conductance of *abi1* plants is approximately twice as high as in the L*er* wild‐type (Merilo *et al*., 2013) and stomata are approximately twice as wide open (Fig. 7a). As expected, stomata of *abi1‐1* did not close after stimulation with ABA (Fig. 7a). However, despite the ABA insensitivity, stomata of *abi1‐1* were still responsive to flg22 (Fig. 7a). This suggests that the guard cell signaling pathways of flg22 and ABA meet at OST1, downstream of the ABA receptor complex that includes ABI1 (Cutler *et al*., 2010) and the flg22 receptor complex with FLS2 (Boller & Felix, 2009).

## Discussion

In guard cells of intact *Arabidopsis* leaves, the bacterial MAMP flg22 triggers rapid stomatal closure after a lag time of *c*. 15 min. This response could be linked to the activation of the two anion channels, SLAC1 and SLAH3; loss of these S‐type anion channels renders stomata flg22‐insensitive. Anion channels release anions into the guard cell wall, thereby depolarizing the plasma membrane (Roelfsema *et al*., 2012; Kollist *et al*., 2014). Owing to this depolarization, guard cells also extrude K^+^ through K^+^ efflux channels, they lose osmolytes and shrink, and so the stomatal pores close. S‐type anion channels thus seem to function as master switches for MAMP‐induced stomatal closure (Roelfsema *et al*., 2012).

### Activation of S‐type anion channels by OST1, CPKs, and CIPKs

Heterologous expression experiments in *Xenopus laevis* oocytes revealed that OST1 activates the SLAC1 anion channel by phosphorylation of its N‐terminal domain (Geiger *et al*., 2009; Lee *et al*., 2009; Vahisalu *et al*., 2010). Within this domain, phosphorylation of Ser120 is essential for channel activation, but is not sufficient (Geiger *et al*., 2009). In the *slac1‐7* mutant, OST1 can no longer activate SLAC1 as a result of a Ser120Phe mutation (Vahisalu *et al*., 2010). The *slac1‐7* mutation causes reduced stomatal closure in response to several stimuli, including high atmospheric CO_2_ concentrations, low relative air humidity, darkness, and ozone (Merilo *et al*., 2013). This suggests that phosphorylation of SLAC1 by OST1 is relevant for its activation, but it is unlikely to be the only mechanism that regulates this S‐type anion channel in guard cells. Most likely, SLAC1 is activated in guard cells by at least two signaling mechanisms, a ‘shortcut pathway’ in which OST1 phosphorylates SLAC1, and a second ‘loop pathway’ that involves CPKs (Fig. 7b).

Several lines of evidence point towards an important role for CPKs in regulating stomatal movement. First, several members of this protein family, for example, CPK3, 6, 21 and 23, are capable of activating SLAC1 in the *Xenopus* oocyte expression system (Geiger *et al*., 2010; Brandt *et al*., 2012; Scherzer *et al*., 2012). Moreover, CPK21 is also capable of activating SLAH3, which cannot be activated by OST1 (Geiger *et al*., 2011; Demir *et al*., 2013). These data indicate that CPKs act in a ‘loop pathway’ (Fig. 7b) by which ABA or flg22 can also activate S‐type anion channels. The role of CPKs in stomatal closure is supported by mutants that have lost either CPK6, both CPK3 and 6, or CPK4 and 11 (Mori *et al*., 2006; Zhu *et al*., 2007; Ye *et al*., 2013). In the *cpk6* loss‐of‐function mutants, guard cells were less responsive to a yeast elicitor (YEL), whereas *cpk3/6* or *cpk4/11* double mutants had impaired responses to ABA.

In contrast to the results with single and double *cpk* loss‐of‐function mutants, nanoinfusion experiments revealed neither the loss of ABA nor flg22 sensitivity in the *cpk3/5/6/11* quadruple mutant (Fig. 6). These contrasting results may be explained by the existence of several mechanisms through which OST1 can activate SLAC1 (Fig. 7b). Whereas OST1 might signal through distinct CPKs at a given growth phase of plants, direct activation of SLAC1 by OST1 can occur at different stages. Moreover, SLAC1 and SLAH3 can also be activated by the CBL‐interacting protein kinase (CIPK) 23, which interacts with the Calcineurin B‐like (CBL) proteins 1 and 9 (Maierhofer *et al*., 2014) (Fig. 7b). The conditions under which these alternative pathways downstream of OST1 are targeted by flg22 or ABA requires further studies.

### Role of Ca^2+^ and ROS signals in MAMP‐induced stomatal closure

Elevation of the cytosolic free Ca^2+^ concentration results in a rapid activation of S‐type anion channels in guard cells (Chen *et al*., 2010; Stange *et al*., 2010). Flg22 may therefore stimulate SLAC1 and SLAH3 through these cytosolic Ca^2+^ signals (Fig. 7b). In line with this mechanism, flg22 was shown to trigger a transient elevation of the cytosolic free Ca^2+^ concentration in various cell types, including guard cells (Lecourieux *et al*., 2005; Jeworutzki *et al*., 2010; Ranf *et al*., 2011; Thor & Peiter, 2014). Upon elevation of the cytosolic Ca^2+^ concentration, specific CPKs will be activated depending on their Ca^2+^ affinity, which was shown to vary considerably between the members of the CPK protein family (Geiger *et al*., 2010; Boudsocq *et al*., 2012). Some CPKs do not show any regulation by Ca^2+^, which suggests that these proteins are regulated via Ca^2+^‐independent mechanisms.

In addition to the activation of anion channels, flg22‐stimulated CPKs can also activate plasma membrane RBOH proteins that produce ROS in the extracellular compartment (Felix *et al*., 1999; Ranf *et al*., 2011; Dubiella *et al*., 2013). In mesophyll cells of *Arabidopsis*, the flg22‐i nduced ROS production depends on a subset of CPKs, including CPK5, 6 and 11 (Boudsocq *et al*., 2010; Dubiella *et al*., 2013). Consistently, we found that flg22‐triggered ROS production is absent in the *cpk3/5/6/11* quadruple mutant. Recently, the cytosolic Botrytis‐induced kinase 1 (BIK1), which is activated by the FLS2–BAK1 receptor complex, was reported to activate RBOH*D* in a Ca^2+^‐independent manner (Kadota *et al*., 2014; Li *et al*., 2014). At this point it is unclear how the BIK1‐ and CPK‐dependent pathways are interconnected. Despite the absence of ROS signals in mesophyll cells of *cpk3/5/6/11*, flg22 still evoked a depolarization, indicating that anion channel activation in this cell type is independent of ROS signaling.

In guard cells of *Arabidopsis*, ABA evokes the production of ROS by RBOH proteins, which suggests a similar response to that triggered by flg22 in mesophyll cells (Kwak *et al*., 2003). However, the ROS production of guard cells evoked by YEL does not depend on *At*RBOH*D* and *F* and thus guard cells seem to possess an alternative mechanism of producing ROS (Khokon *et al*., 2010). Flg22‐induced ROS have not yet been measured in guard cells, and inconsistent results were obtained for stomatal responses of NADPH oxidase mutants. Whereas nanoinfusion of flg22 triggered stomatal closure in the *AtrbohD/F* double mutant (Fig. 6d), this response was absent in submerged seedlings and epidermal peels of *Atrboh*D (Macho *et al*., 2012; Li *et al*., 2014). Likewise, stomatal closure in epidermal strips was found to depend on AtRBOH*D* and *F* (Kwak *et al*., 2003), whereas stomata of the *AtrbohD/F* double mutant still responded to nanoinfusion of ABA (Fig. 6d). Comparison of these sets of data suggests that the requirement for NADPH oxidases to close stomata depends on the experimental conditions. Consequently, the role of ROS signals in the regulation of guard cell anion channels needs further attention.

### Signaling pathway upstream of OST1

In contrast to the similarities in flg22 and ABA signaling downstream of OST1, the upstream perception mechanisms are very different. Whereas the PYR/PYL/RCAR ABA receptors are located in the cytosol (Ma *et al*., 2009; Park *et al*., 2009), flg22 is perceived by the FLS2 receptor in the plasma membrane (Figs 6d, 7b). Upon flg22 binding, the FLS2 receptor interacts with BAK1 and homologous SERKs (Chinchilla *et al*., 2007, 2009; Roux *et al*., 2011; Sun *et al*., 2013). These flg22‐stimulated receptor complexes interact with several receptor‐like cytoplasmic kinases that are likely to forward the MAMP signal to activate OST1 in guard cells (Fig. 7b) (Lu *et al*., 2010; Shi *et al*., 2013; Ranf *et al*., 2014).

The cytosolic ABA receptor proteins interact and inhibit type 2C protein phosphatases (PP2C), including ABI1 (Ma *et al*., 2009; Park *et al*., 2009). Owing to the PP2C inhibition, OST1 becomes activated in the presence of ABA and provokes stomatal closure (Cutler *et al*., 2010; Raghavendra *et al*., 2010). The point mutation in *abi1‐1*, which results in ABI1^G180D^, prevents its interaction with ABA receptors (Ma *et al*., 2009; Park *et al*., 2009). This causes ABI1^G180D^ to remain active in the presence of ABA and thus prevents ABA responses. However, stomata of *abi1‐1* still close in response to flg22, which suggests that activation of OST1 by flg22 is not affected by the inability of ABA receptors to bind to ABI1. Previously, it was shown that activation of OST1 by an osmotic shock is also unaffected in *abi1‐1* seedlings (Vlad *et al*., 2010). This suggests that OST1 can be activated by mechanisms that do not depend on ABI1 interaction with the ABA receptors.

### OST1, a central hub in guard cell signaling

The OST1 protein kinase seems to be a key regulator for triggering stomatal closure (Mustilli *et al*., 2002; Roelfsema *et al*., 2012). Its role has been studied most intensively with respect to ABA responses, but OST1 is also required for stomatal closure in response to other stimuli, such as CO_2_ and ozone (Xue *et al*., 2011; Merilo *et al*., 2013). Here we show that OST1 is essential for flg22‐dependent stomatal closure, as was previously reported by Melotto *et al*. (2006). By contrast, Montillet *et al*. (2013) found that stomata of *ost1‐2* close in response to flg22. The difference in results between these studies is probably caused by differences in the experimental conditions. In this study, we stimulated stomata by nanoinfusion of a solution with 20 nM flg22 into intact leaves, whereas Montillet *et al*. (2013) and Melotto *et al*. (2006) applied 10 μM flg22 to stomata in isolated epidermal peels. In addition to the higher MAMP concentration, Montillet *et al*. (2013) also grew plants at a light: dark cycle of 18 : 6 h, while our study and that of Melotto *et al*. (2006) used a light: dark cycle of 12 : 12 h. Our study showed that stomatal closure triggered by 20 nM flg22 depends on OST1. It is likely that such low MAMP concentrations are more relevant for innate immunity and will thus trigger stomatal closure during bacterial infections.

It is likely that the mechanism causing MAMP‐dependent activation of S‐type channels is conserved in seed plants, because in a study with tobacco Bright Yellow‐2 (BY‐2) cells expressing the *Arabidopsis* SLAC1 channel, Cl^−^ extrusion was triggered by the tobacco‐specific MAMP cryptogein (Kurusu *et al*., 2013). This suggests that the tobacco defense signaling pathway in BY‐2 cells is capable of activating the *Arabidopsis* SLAC1 anion channel. Even though the activation of anion channels is common in plant cell immunity, the signaling pathways leading to this response are likely to vary between cell types. Whereas OST1 is highly expressed in guard cells and plays a key role in stomatal closure, ABA responses in other cell types are dependent on two additional OST1‐homologous protein kinases, SnF1 Related Kinase2.2/2D (SnRK2.2/2D) and SnRK2.3/2I (Fujii & Zhu, 2009; Fujita *et al*., 2009).

Insights into cell type‐specific differences and commonalities in early pathogen defense responses could help to improve plant pathogen resistance. In this respect, the OST1 protein kinase is an interesting target for plant breeding, as it integrates signals related to abiotic conditions, such as humidity and the intracellular CO_2_ concentration, and biotic signals, such as the presence of MAMPs. Thus, modifying this hub can potentially improve both drought and pathogen tolerance. Therefore a better understanding of OST1 functions will not only lead to insights into the mechanisms that control transpiration, but could also lead to new strategies to improve crop yield.

## Supporting information

Please note: Wiley Blackwell are not responsible for the content or functionality of any supporting information supplied by the authors. Any queries (other than missing material) should be directed to the *New Phytologist* Central Office.

**Movie S1** Movie of stomatal closure induced by nanoinfusion of 20 nM flg22.

**Fig. S1** Sensitivity of platinum‐iridium disc electrode to H_2_O_2_ production by mesophyll tissue.
**Fig. S2** Time‐dependent stomatal movement induced by nanoinfusion of control solution, 10 μM ABA, or 20 nM flg22 in selected accessions and mutants.
