# Supplementary material for: Guard cell SLAC1‐type anion channels mediate flagellin‐induced stomatal closure
Source: New Phytol. 2015 Apr 30;208(1):162–73. doi: 10.1111/nph.13435 (PMC4949714; doi:10.1111/nph.13435)
Supplement: Supplementary file 2 — Fig. S1 Sensitivity of platinum‐iridium disc electrode to H2O2 production by mesophyll tissue. Fig. S2 Time‐dependent stomatal movement induced by nanoinfusion of control solution, 10 μM ABA, or 20 nM flg22 in selected accessions and mutants. [file NPH-208-162-s002.pdf]

New Phytologist **Supporting Information Figs S1 & S2**

Article title: Guard cell SLAC1-type anion channels mediate flagellin-induced stomatal closure

Authors: Aysin Guzel Deger, Sönke Scherzer, Maris Nuhkat, Justyna Kedzierska, Hannes Kollist, Mikael Brosché, Serpil Unyayar, Marie Boudsocq, Rainer Hedrich and M. Rob G. Roelfsema

Article acceptance date: 29 March 2015

The following Supporting Information is available for this article:

Fig. S1 Sensitivity of platinum-iridium disc electrode to H_2O_2 production by mesophyll tissue.

Fig. S2 Time-dependent stomatal movement induced by nanoinfusion of control solution, 10 µM ABA, or 20 nM flg22 in selected accessions and mutants.

Movie S1 Movie of stomatal closure induced by nanoinfusion of 20 nM flg22 (separate .avi file).

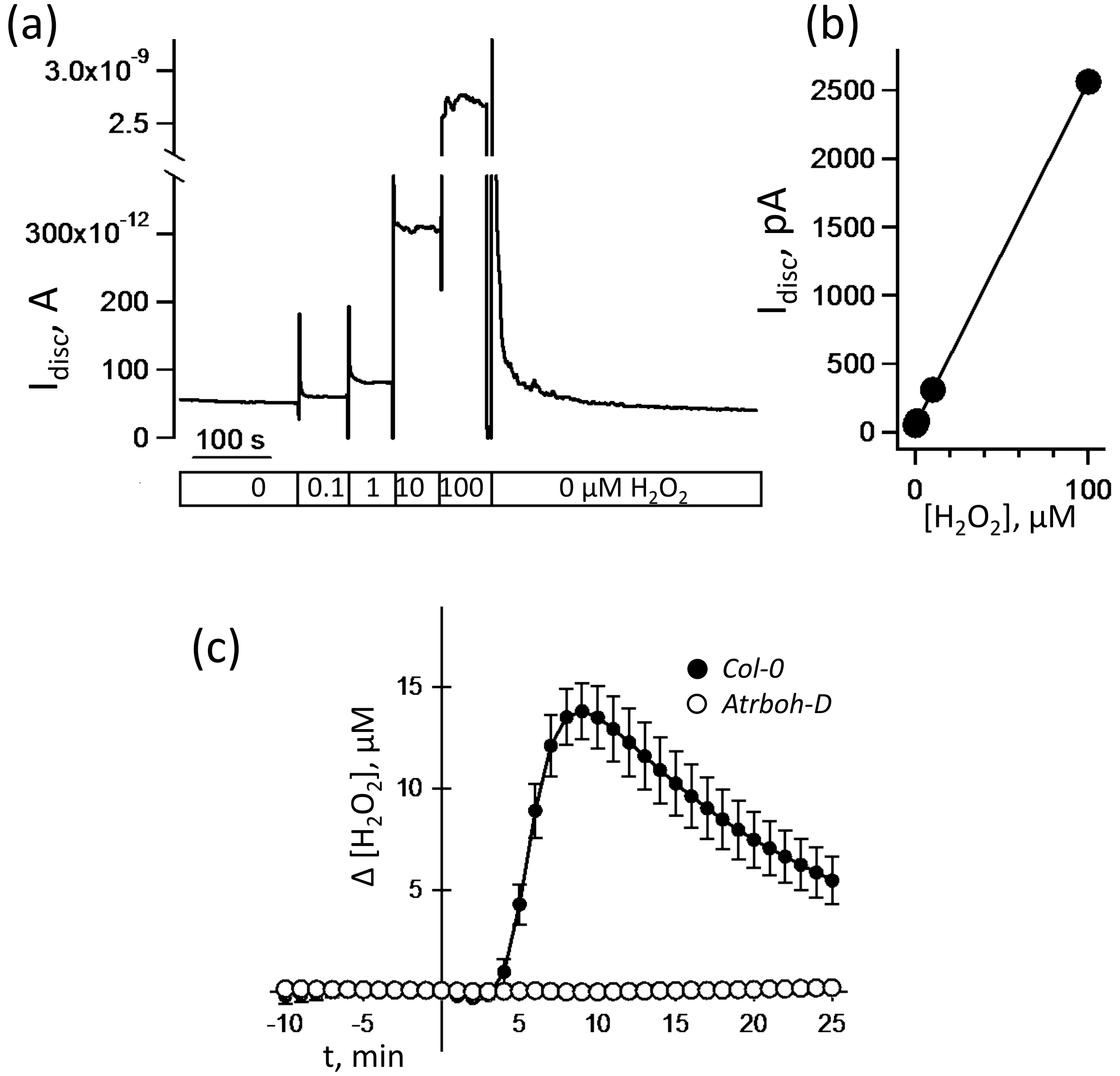

Fig. S1 Sensitivity of platinum-iridium disc electrode to hydrogen peroxide (H_2O_2) production by mesophyll tissue. (a) Calibration of the platinum-iridium electrodes with H_2O_2 at concentrations ranging from 0.1 to 100 µM, as given in the bar below the current trace. An increase in H_2O_2 concentration causes a rapid and reversible increase in current. (b) A linear relation was obtained between the H_2O_2 concentration and the current measured with the platinum-iridium disc electrode. (c) Flg22-induced H_2O_2 production of mesophyll tissue measured with the microdisc-electrode. H_2O_2 production was measured in Col-0 (closed circles) and the *Atrboh*-D mutant (open circles). At t = 0, 20 nM Flg22 was added to the bath solution, error bars represent ± SE of 11 (Col-0) or 3 (*Atrboh*-D) experiments.

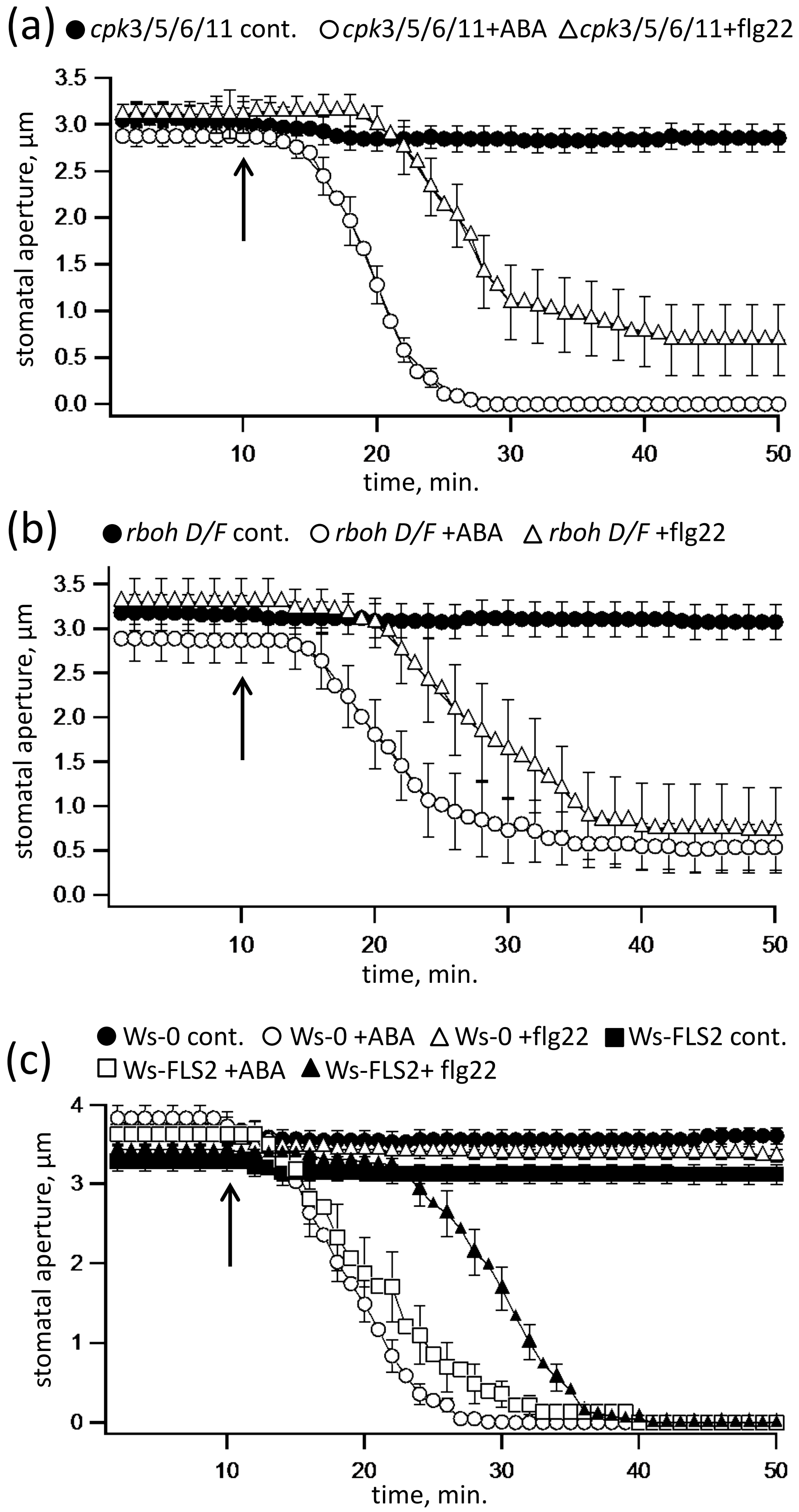

Fig. S2 Time-dependent stomatal movement induced by nanoinfusion of control solution, 10

µM ABA, or 20 nM flg22 in selected accessions and mutants. (a) Stomata of the *cpk3/5/6/11* quadruple mutant stimulated with control solution (closed circles, *n* = 11), 10 µM ABA (open circles, *n* = 18), or 20 nM flg22 (open triangles, *n* = 16). (b) Stomata of the *rbohD/F* double mutant stimulated with control solution (closed circles, *n* = 10), 10 µM ABA (open circles, *n* = 12), or 20 nM flg22 (open triangles, *n* = 11), and (c) Wassilewskija (Ws-0) wild type stomata stimulated with control solution (closed circles, *n* = 11), 10 µM ABA (open circles, *n* = 12), or 20 nM flg22 (open triangles, *n* = 11), as well as FLS2 transformed Ws-0 (Zipfel *et al.*, 2004) stimulated with control solution (closed squares, *n* = 10), 10 µM ABA (open squares, *n* = 10) or 20 nM flg22 (closed triangles, *n* = 12). Data are given as average values of 10–18 stomata of at least three independent experiments. Error bars represent ± SE, arrows indicate the time point of nanoinfusion.

Reference

Zipfel C, Robatzek S, Navarro L, Oakeley EJ, Jones JDG, Felix G, Boller T. 2004. Bacterial disease resistance in *Arabidopsis* through flagellin perception. *Nature* **428:** 764–767.

Movie S1 (separate .avi file) Stomatal closure induced by nanoinfusion of 20 nM flg22. Data are from the same stoma in the abaxial epidermis of an *Arabidopsis* leaf as shown in Fig. 1(b). Images were obtained every 30 s. Nanoinfusion through a neighboring stoma (not visible in the image) occurred after 10 min and is evident by a change in transparency of the epidermis, because of the solution infused into the intercellular space.
